# Cohort retention in a pandemic response study: lessons from the SARS-CoV2 Immunity & Reinfection Evaluation (SIREN) study

**DOI:** 10.1186/s12874-025-02469-6

**Published:** 2025-01-30

**Authors:** Anna Howells, Katie Munro, Sarah Foulkes, Atiya Kamal, Jack Haywood, Sophie Russell, Dominic Sparkes, Erika Aquino, Jennie Evans, Dale Weston, Susan Hopkins, Jasmin Islam, Victoria Hall

**Affiliations:** 1 United Kingdom Health Security Agency (UKHSA), London, UK; 2 School of Social Sciences, Birmingham City University, Birmingham, UK; 3 British Society for Immunology, 9 Appold Street, London, EC2A 2AP UK

**Keywords:** Cohort retention, Pandemic response, COVID-19, Participant involvement, Participant engagement, SIREN, SARS-CoV-2, Coronavirus

## Abstract

**Background:**

SIREN is a healthcare worker cohort study aiming to determine COVID-19 incidence, duration of immunity and vaccine effectiveness across 135 NHS organisations in four UK nations. Conducting an intensive prospective cohort study during a pandemic was challenging. We designed an evolving retention programme, informed by emerging evidence on best practice. This included applying a multifactorial approach, and considering strategies for barrier reduction, community building, follow-up, and tracing. We utilised participant engagement tools underpinned by our Participant Involvement Panel (PIP) and here we evaluate cohort retention over time and identify learnings.

**Methods:**

A mixed method evaluation of cohort retention in 12 and 24-month follow-up (June 2020 – March 2023). We described cohort retention by demographics and site, using odds ratios from logistic regression. Withdrawal reasons during this time were collected by survey. We collected participant feedback via cross-sectional online survey conducted October – November 2022, utilising a behavioural science approach. We conducted two focus groups with research teams in February 2023 and conducted thematic analysis exploring cohort retention challenges and facilitators.

**Results:**

37,275 (84.7%) participants completed 12-months of follow-up. Of 14,772 participants extending their follow-up to 24 months, 12,635 (85.5%) completed this. Retention increased with age in the 12 (55–64 years vs < 25 years OR = 2.50; 95% CI: 2.19–2.85; *p* < 0.001) and 24-month (> 65 years vs < 25 years OR = 2.92; 95% CI: 1.78–4.88; *p *< 0.001) cohorts. Retention was highest in the Asian and Black ethnic groups compared to White in the 12 (OR = 1.38; 95% CI: 1.23–1.56; *p* < 0.001, and OR = 1.64; 95% CI: 1.30–2.08; *p* < 0.001) and 24-month (OR = 1.78; 95% CI: 1.42–2.25; *p* < 0.001, and OR = 2.12; 95% CI: 1.41–3.35; *p* < 0.001) cohort. Among participants withdrawing, the median time in follow-up at withdrawal was 7 months (IQR: 4–10 months) within the 12-month cohort and 19 months within the 24-month cohort (IQR: 16–22 months). The top three reasons for participant withdrawal were workload, leaving site employment and medical reasons. Themes identified from focus-groups included: the need to monitor and understand participant motivation over time, the necessity of inclusive and comprehensive communication, the importance of acknowledging participant contributions, building collaboration with local research teams, and investing in the research team skillset.

**Conclusion:**

Participant retention in the SIREN study remained high over 24-months of intensive follow-up, demonstrating that large cohort studies are feasible as a pandemic research tool. Our evaluation suggests it is possible to maintain an engaged cohort of healthcare workers (HCWs) during an acute pandemic response phase. The insights gained from this population group are important, as a highly exposed group fulfilling an essential pandemic response and patient care function. The success of the cohort study overall, as well as the specific population group retained, offer useful insight for pandemic preparedness planning and healthcare worker studies.

**Supplementary Information:**

The online version contains supplementary material available at 10.1186/s12874-025-02469-6.

## Background

In recent years a growing evidence base of cohort retention strategies for longitudinal studies has developed [1–5]. However, heterogeneity in approach is common given the unique and study-specific context of research questions, methodology, settings and population groups [1, 2]. Nevertheless, common cohort retention strategies have emerged in addition to agreement about the resources needed to deliver these strategies. A recent systematic review of 95 longitudinal cohort studies [1] highlights four key cohort retention strategies. The first of these is barrier reduction, which includes approaches such as flexible data collection methods and engaging a participant sub-sample to evaluate data collection methods. Community building refers to efforts to build a study brand and sharing study results. Strategies to improve follow-up rates include reminders and incentives to complete assessments. Tracing strategies include using locator documents, collecting alternate contact details and using alternative data records to provide updated contact information. In addition, it is common for studies to employ a multifactorial approach to cohort retention [4] to allow for individual participant preference, drawing on numerous cohort retention approaches.

The literature to date has also identified key resources needed to support cohort retention strategies. Common resources identified include dedicated staff who receive training in participant engagement; the ability to collect, store and access data on retention over time; and the capacity to continuously evaluate retention over time [3–5].

Maximising cohort retention can benefit longitudinal studies through reducing administration costs and improving the efficiency of research processes [3]. A high attrition rate can reduce the generalisability of outcomes and the statistical power to detect effects of interest [1] and increases the risk of bias, particularly if those lost to follow-up differ from those retained in the study [3].

The COVID-19 pandemic dramatically changed the health research landscape, presenting new challenges to recruiting, running and retaining participants in longitudinal cohort studies [5]. This is particularly true for studies involving healthcare workers (HCW) as this cohort were at the frontline of the pandemic response, resulting in increased workloads, physical and mental ill health for many [6–8], likely reducing and restricting staff availability or motivation to participate in research studies. It is within this challenging context that the SARS-CoV-2 Immunity and Reinfection Evaluation (SIREN) study was established and designed an evolving and multifactorial cohort retention programme, incorporating approaches to participant engagement and involvement.

SIREN is a prospective multicentre cohort study established in 2020 to evaluate the immune response to SARS-CoV-2 following infection and vaccination in UK healthcare workers [9]. Study participants are National Health Service (NHS) staff working at healthcare organisations in a clinical setting where patients are present, which includes administrative, executive and support staff, resulting in a broad representation of the NHS workforce [9].

The aim of this study is to describe and evaluate the SIREN study approach to cohort retention in the 12 and 24-month cohort, analysing corresponding retention figures and participant and NHS site feedback to inform lessons for future studies.

## Methods

### SIREN cohort retention approach

**Fig. 1:**
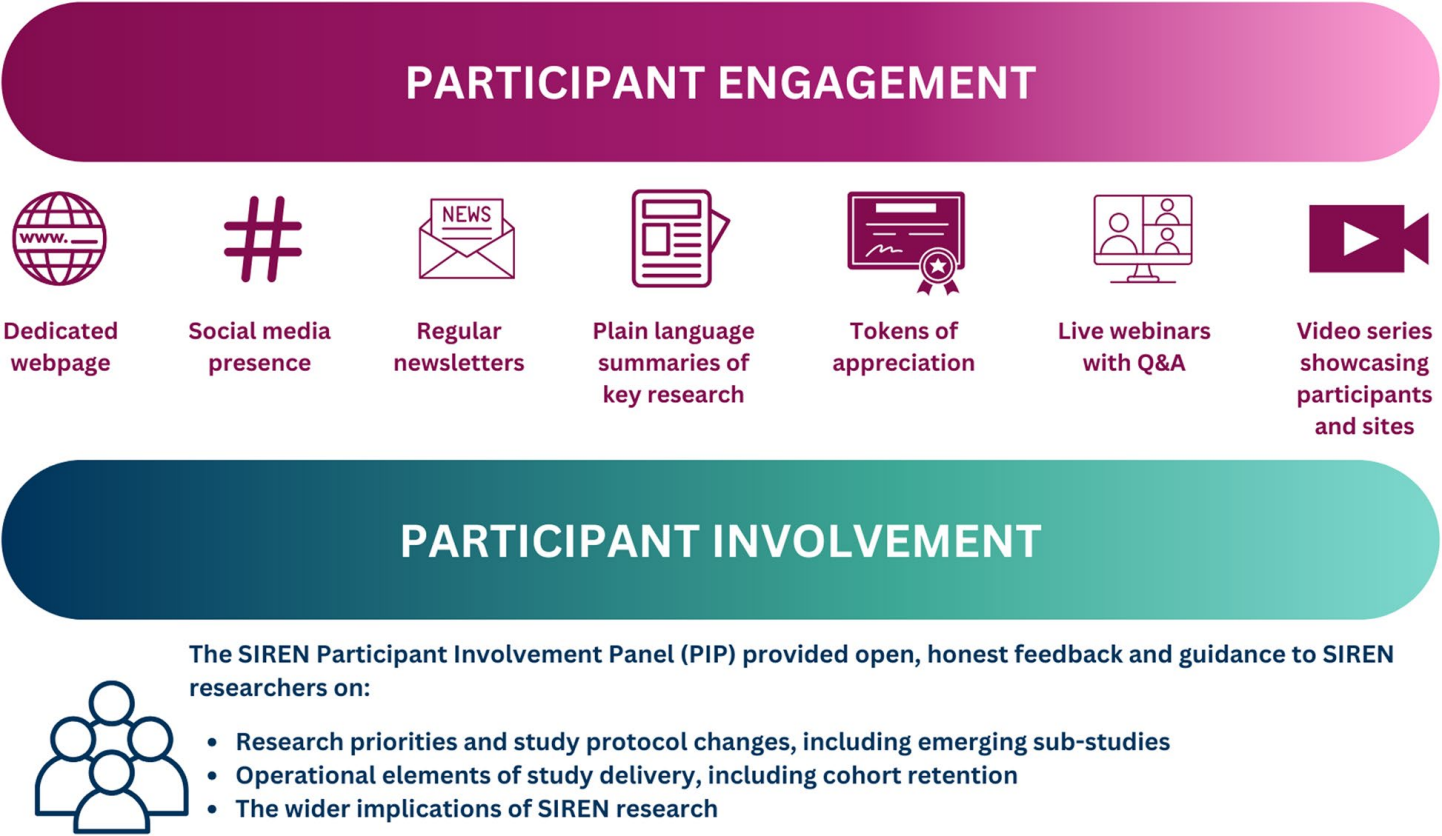
The SIREN approach to cohort retention includes a focus on participant engagement and involvement

The SIREN approach to cohort retention included a focus on participant engagement and involvement (Fig. 1). To engage participants, SIREN produced regular digital newsletters, hosted interactive webinars and provided a dedicated study website. A key SIREN participant involvement tool was the Participant Involvement Panel (PIP). The PIP was a panel of participants who met regularly and provided guidance and feedback to SIREN researchers on key research priorities, proposed study changes and strategies for maximising participant engagement [10]. The full learning and impact of the PIP is reported elsewhere [10].

### Study design

A mixed-method process and outcome evaluation of our evolving cohort retention programme involving qualitative and quantitative analysis nested within a national, multicentre prospective cohort study.

### Study population

SIREN participants were healthcare workers and support staff aged 18 years and over who worked at enrolment at any of the 135 NHS hospital or health board SIREN sites across the United Kingdom. SIREN study participants and SIREN site research teams (individuals involved in running the SIREN study at participating organisations) took part in this evaluation.

### Outcomes

The primary outcome was the proportion of participants completing their scheduled follow-up period. Secondary outcomes included reasons for withdrawal, trends in withdrawals over time, participant engagement and participant experience of their study involvement.

### Data sources

#### Enrolment survey

Participant demographic data (including age, ethnic group, job role and geographic location) were collected on all SIREN participants via the online enrolment survey. The enrolment survey was built and hosted using SnapSurveys TM software v11 [11].

#### Withdrawal survey

Participants who chose to withdraw prior to the completion of their scheduled follow-up period were required to fill out an online survey with eight categories of withdrawal reasons provided, in addition to an “other” free-text option to expand on their reasoning.

#### Participant feedback survey

A cross-sectional survey to capture participant feedback was conducted in October–November 2022. A behavioural insights approach was used to develop survey questions using the Capability, Opportunity, Motivation, Behaviour (COM-B) model [12]. The COM-B model recognises that for any behaviour to be enacted individuals must have the capability, opportunity and motivation to do so, beyond anything that is in competition with the desired behaviour [13]. The feedback survey included distinct questions capturing an individual’s capability, opportunity and motivation to continue participating in SIREN study (Appendix 1).

The survey was sent to participants directly via email or text (dependent on stated communication preference), highlighted in subsequent issues of the SIREN digital newsletter and referenced at monthly site meetings for onward distribution by research teams. Participants were sent a reminder about the survey a week before the closing date.

#### Focus groups

Two focus groups with SIREN site research teams took place in February 2023 to form part of study evaluation. Focus groups attendees were asked to describe how they had managed participant retention at their site, the challenges they had encountered and what helped with retention.

Focus groups were advertised to SIREN site research teams via regular monthly meetings and via digital newsletters. Each focus group attendee was offered an honorarium in line with National Institute for Health and Care Research (NIHR) guidance [14]. Attendees for focus groups were selected to represent as broad a range of SIREN sites as possible with consideration of demographic groups such as gender, age, ethnicity and staff group.

Focus groups were held over Microsoft Teams [15] and facilitated by two external partners with expertise in qualitative research to facilitate an open and honest conversation about positive and negative experiences of taking part in SIREN. Focus groups ranged from 64 to 116 min and were recorded using the inbuilt Microsoft Teams recording function [15]. Interview recordings were transcribed verbatim, anonymised, and checked for accuracy by the research team.

### Data analysis

#### Retention

The SIREN cohort retention approach was compared to frequently cited strategies in the literature to identify similarities and highlight gaps.

We used completion of 12 and 24-months of follow-up on or before 31 March 2023, as the primary outcome of our statistical analysis. We described cohort retention in the 12 and 24-month cohorts overall, and by age group, gender, ethnic group, staff group and site size using odds ratios from logistic regression.

#### Withdrawals

Using responses to the withdrawal survey, we reported the frequency of reasons for withdrawal among participants in the 12 and 24-month cohorts and highlighted trends in withdrawal reasons over time. Participant withdrawals were analysed by both person-time contributed to the study and by calendar month.

#### Engagement

We used the average number of follow-up surveys completed by participants who completed the first 12 months of follow-up as an outcome for study engagement.

#### Feedback

Participant feedback survey responses were analysed by demographic groups and key findings reported. Feedback survey responses were analysed using the COM-B model [12] as a framework to understand whether participants had the capability, opportunity and motivation necessary to undertake the desired behaviour (continued participation in the SIREN study).

Participant feedback was also collected in the withdrawal survey, in a free-text field. Responses were first categorised as positive, negative or neutral, and then analysed using the COM-B framework as above to understand whether withdrawal was due to factors influencing an individual’s capability, opportunity or motivation.

#### Focus groups

Transcripts were analysed inductively using thematic analysis. This included familiarisation with data by reading and re-reading transcripts, generating initial codes by identifying key features based on participant responses, and identifying themes by making connections between codes which were grouped into categories. The themes were then reviewed, refined, named, and written up. Coding of each transcript was conducted independently by two external researchers who met regularly to compare coding for each transcript and to form connections, resulting in themes that were included in the analysis.

## Results

### Participant retention

The SIREN approach to cohort retention was informed by key components of good practice identified in the literature, as described in Table 1. SIREN provided key resources for the cohort retention programme, including dedicated staff, retention tracked over time via monthly figures, and ongoing evaluation via tools such as the withdrawal survey, inbox monitoring and regular PIP meetings. In terms of key cohort retention strategies, SIREN developed approaches to barrier reduction (via consistent leadership and the PIP), community building (via branded study communications including newsletters and thank you messages) and follow-up (options for SMS or email communication). SIREN does not undertake the tracing strategies as described in the literature, although in addition to direct participant contact SIREN has utilised site research teams as a further avenue for communication with participants.

**Table 1:** Key cohort retention strategies identified in the literature with comparison to the SIREN study approach

Category	Included in SIREN approach	Detail
**Resources **[3]		
Dedicated staff and training [3]	✓	• SIREN recruited research staff with specific responsibilities for cohort retention • Collaboration with British Society for Immunology to run the SIREN PIP • Formal training in retention methodology not widely available
Retention tracked over time	✓	• Monthly retention figures produced with trends by age group, gender, ethnic group, staff group, region and site size
Ongoing evaluation	✓	• Retention figures regularly reviewed and disseminated to SIREN research team and sites • Monitoring and exploration of reasons for withdrawal via the withdrawal survey • Central SIREN inbox management and monitoring • Close out process offered to all participating sites which includes opportunity for research teams to provide feedback • Regular meetings with the PIP who provide feedback on proposed study changes, participant engagement activities general feeling among participants
**Strategies **[2, 4]		
Barrier reduction *Includes flexible methods of data collection, offering site and home visits, consistent research team members, engaging a participant sub-sample to evaluate data collection methods*	✓	• SIREN has maintained the same leadership for the duration of the study who are visible to participants via newsletters, webinars and videos • SIREN data collection includes regular online surveys, serology samples, PCR and LFD tests. Participant schedules vary depending on their study pathway and it is common for SIREN site teams to offer flexibility for sample collection / drop off • The PIP provides a forum for feedback on study approaches including data collection
Community building *Includes thank you messages, creating a recognisable study brand and the sharing of study results*	✓	• SIREN logo is included on all study material • SIREN has provided regular digital newsletters which includes key study updates and findings and thank you messages to acknowledge key study milestones • SIREN has provided a dedicated webpage, hosts live webinars for participants and has created a video series to showcase the study • SIREN has provided tokens of appreciation for participants including stickers, certificates and mugs and badges for site teams
Follow-up *Includes cash or voucher incentives, phone calls, SMS, mail and email reminders, house visits*	✓	• SIREN has provided voucher incentives as part of prize draws • SIREN participants can select to be contacted via SMS or email
Tracing *Includes collecting details of alternative contact persons, using public or non-public records to find updated contact information and use of locator documents*	X	• SIREN does not rely on alternative records to find updated contact information • Site research teams play an important role in monitoring participant follow-up

Using data from the enrolment and withdrawal surveys we found that of the 44,543 participants recruited into 12 months of follow-up at 135 SIREN sites, 37,725 (84.7%) completed their 12-months of follow-up (Table 2). Extension into a second year of follow-up was offered at 87 SIREN sites, and 14,772 participants at these sites consented to extend their follow-up to 24 months. Of these, 12,635 (85.5%) participants completed their 24-month follow-up (Table 3).

**Table 2:** Cohort retention in the 12-month cohort, by demographics and SIREN site size

Characteristic	Number of participants consented to 12 months	Completed 12 months n (%)	OR	95% CI	*p*-value
**Gender**					
Female	36,852	31,348 (85.1)	Ref	Ref	Ref
Male	7,212	6,245 (86.6)	1.13	1.04, 1.22	0.004
Other	65	56 (86.2)	1.33	0.66, 3.06	0.5
**Age group**					
Under 25	1,741	1,333 (76.6)	Ref	Ref	Ref
25 to 34	8,929	7,140 (80.0)	1.18	1.04, 1.33	0.009
35 to 44	10,773	9,299 (86.3)	1.86	1.64, 2.10	< 0.001
45 to 54	13,082	11,596 (88.6)	2.32	2.05, 2.63	< 0.001
55 to 64	8,540	7,617 (89.2)	2.50	2.19, 2.85	< 0.001
Over 65	688	608 (88.4)	2.34	1.81, 3.05	< 0.001
**Ethnicity**					
White	38,680	32,858 (84.9)	Ref	Ref	Ref
Asian	3,238	2,861 (88.4)	1.38	1.23, 1.56	< 0.001
Black	885	797 (90.1)	1.64	1.30, 2.08	< 0.001
Mixed Race	680	570 (83.8)	1.01	0.82, 1.26	0.9
Other Ethnic Group	557	488 (87.6)	1.23	0.95, 1.61	0.12
Prefer not to say	89	75 (84.3)	0.79	0.46, 1.48	0.4
**Staff type**					
Nursing	14,904	12,762 (85.6)	Ref	Ref	Ref
Administrative/Executive	6,653	5,609 (84.3)	0.84	0.77, 0.91	< 0.001
Doctor	5,219	4,504 (86.3)	1.01	0.92, 1.12	0.8
Healthcare Assistant	3,647	3,078 (84.4)	0.91	0.82, 1.01	0.088
Healthcare Scientist	2,516	2,130 (84.7)	1.00	0.89, 1.13	> 0.9
Student	1,507	1,297 (86.1)	1.02	0.88, 1.20	0.8
Therapist	1,815	1,503 (82.8)	1.01	0.88, 1.16	0.9
Midwife	954	829 (86.9)	1.19	0.98, 1.46	0.092
Pharmacist	930	784 (84.3)	1.06	0.88, 1.29	0.6
Estates/Porters/Security	703	607 (86.3)	0.99	0.78, 1.25	0.9
Other	5,281	4,546 (86.1)	1.07	0.98, 1.18	0.2
**Site size**					
Small ≤ 200	5,203	4,445 (85.4)	Ref	Ref	Ref
Medium 201–800	32,118	27,469 (85.5)	1.00	0.91, 1.09	> 0.9
Large > 800	7,222	5,811 (80.5)	0.75	0.68, 0.84	< 0.001
**Total**	**44,543**	**37,725 (84.7)**

**Table 3:** Cohort retention in the 24-month cohort, by demographics and SIREN site size

Characteristic	Number of participants consented to 24 months	Completed 24 months n (%)	OR	95% CI	*p*-value
**Gender**					
Female	12,379	10,577 (85.4%)	Ref	Ref	Ref
Male	2,325	2,031 (87.4%)	1.15	1.00, 1.32	0.054
Other	20	18 (90.0%)	1.33	0.37, 8.44	0.7
**Age group**					
Under 25	233	176 (75.5)	Ref	Ref	Ref
25 to 34	1,996	1,584 (79.4)	1.23	0.89, 1.69	0.2
35 to 44	3,562	3,022 (84.8)	1.80	1.30, 2.45	< 0.001
45 to 54	5,118	4,540 (88.7)	2.58	1.87, 3.51	< 0.001
55 to 64	3,508	3,072 (87.6)	2.34	1.69, 3.19	< 0.001
Over 65	259	232 (89.6)	2.92	1.78, 4.88	< 0.001
**Ethnicity**					
White	13,009	11,071 (85.1)	Ref	Ref	Ref
Asian	965	875 (90.7)	1.78	1.42, 2.25	< 0.001
Black	317	293 (92.4)	2.12	1.41, 3.35	< 0.001
Mixed Race	217	196 (90.3)	1.71	1.11, 2.78	0.021
Other Ethnic Group	190	167 (87.9)	1.23	0.81, 1.96	0.4
Prefer not to say	26	24 (92.3)	3.62	0.75, 65.1	0.2
**Staff type**					
Nursing	4,944	4,287 (86.7)	Ref	Ref	Ref
Administrative/Executive	2,357	1,968 (83.5)	0.74	0.64, 0.85	< 0.001
Doctor	1,786	1,542 (86.3)	0.86	0.73, 1.02	0.088
Healthcare Assistant	972	845 (86.9)	0.99	0.81, 1.22	> 0.9
Healthcare Scientist	890	763 (85.7)	0.98	0.79, 1.21	0.8
Student	418	366 (87.6)	1.17	0.86, 1.62	0.3
Therapist	617	498 (80.7)	0.76	0.61, 0.95	0.014
Midwife	315	274 (87.0)	1.01	0.73, 1.45	> 0.9
Pharmacist	364	308 (84.6)	0.90	0.67, 1.23	0.5
Estates/Porters/Security	264	235 (89.0)	1.11	0.75, 1.71	0.6
Other	1,797	1,540 (85.7)	0.98	0.84, 1.16	0.8
**Site size**					
Small ≤ 200	1,920	1,642 (85.5)	Ref	Ref	Ref
Medium 201–800	10,851	9,329 (86.0)	1.05	0.91, 1.21	0.5
Large > 800	2,001	1,664 (83.2)	0.86	0.72, 1.02	0.087
**Total**	**14,772**	**12,635 (85.5)**			

**Fig. 2:**
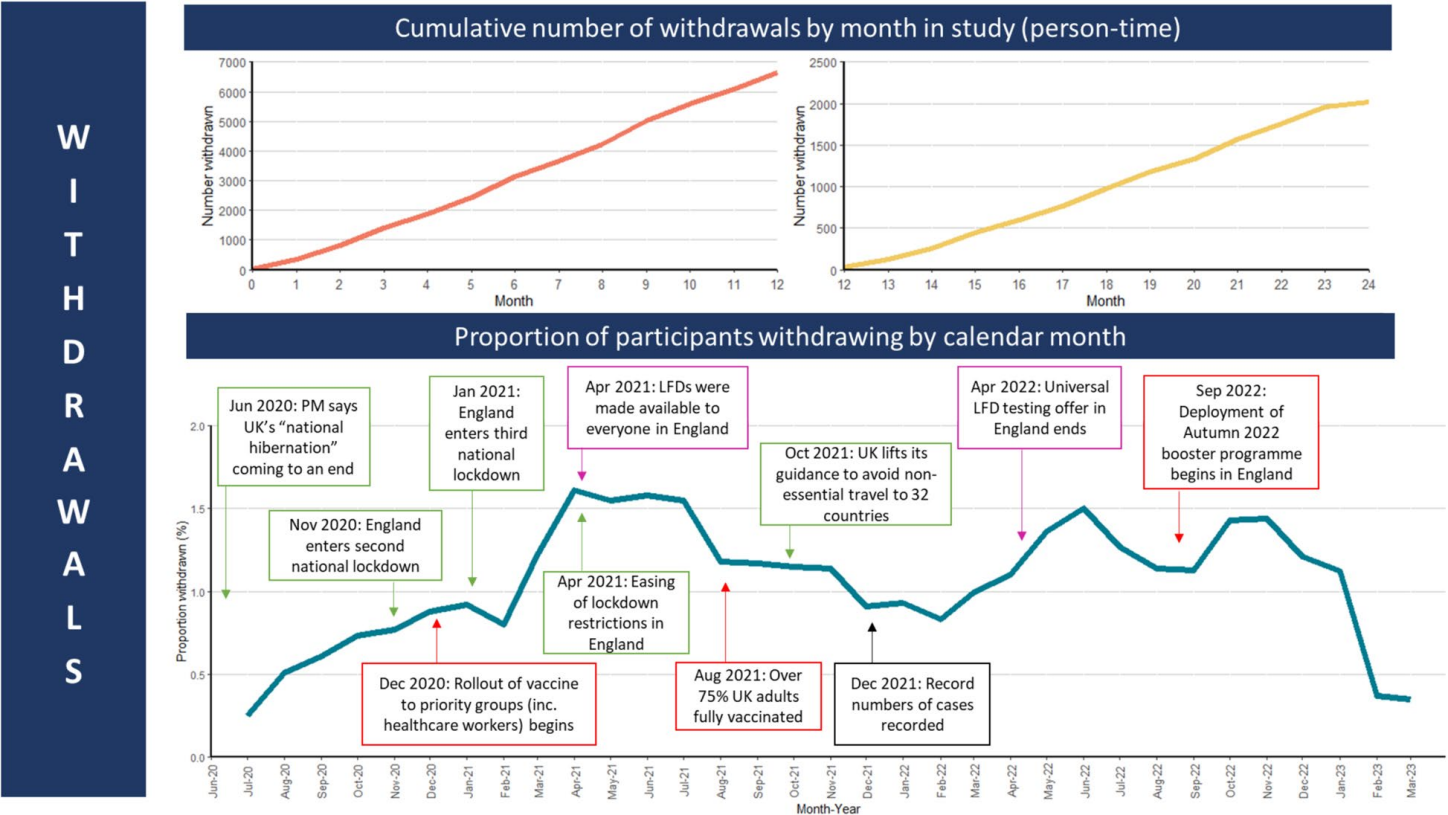
Participant withdrawals by person-time contributed to the study and by calendar month, highlighting external factors that may have influenced retention

Participant retention decreased gradually over time (Fig. 2). Retention in the 12-month cohort was slightly higher among male participants compared to female participants (OR = 1.13; 95% CI: 1.04–1.22; *p* = 0.004). Retention increased with age (55–64 years vs < 25 years OR = 2.50; 95% CI: 2.19–2.85; *p* < 0.001). Retention was highest in the Asian and Black ethnic groups (OR = 1.38; 95% CI: 1.23–1.56; *p* < 0.001, and OR = 1.64; 95% CI: 1.30–2.08; *p* < 0.001, respectively; REF = White). Retention was similarly high across occupational groups (range: 83% to 87%). Retention varied over sites (range: 65.5% to 96.2%) and was higher among smaller sites (85.4% across sites < 200 participants vs. 80.5% sites > 800 participants; *p* < 0.001) (Table 2).

Retention in the 24-month cohort followed a similar trend to the 12-month cohort, with retention increasing with age (> 65 years vs < 25 years OR = 2.92; 95% CI: 1.78–4.88; *p* < 0.001), and highest in the Asian and Black ethnic groups (OR = 1.78; 95% CI: 1.42–2.25; *p* < 0.001, and OR = 2.12; 95% CI: 1.41–3.35; *p* < 0.001, respectively; REF = White) (Table 3). Trends in retention by staff group and site size followed the same trend as the 12-month cohort (Table 3).

### Participant withdrawals

When analysed by person time contributed to the study, participant withdrawals were highest in month 9 of follow-up for the 12-month cohort (791 withdrawals, 11.6%) and in month 21 of follow-up in the 24-month cohort (232 withdrawals, 10.9%). The median time in follow-up for those withdrawing from the 12-month cohort was 7 months (IQR: 4–10 months) and 19 months for those who withdrew from the 24-month cohort (IQR: 16–22 months). By calendar month, the withdrawal rate was highest in April 2021 (717 withdrawals, 1.6% of participants withdrawing), when 90% of participants had received their second vaccine dose (Fig. 2).

The most common reasons for withdrawal in both the 12- and 24-month cohorts were workload commitments (35.6% and 40.9%, respectively), and moving sites/leaving the NHS (18.5% and 22.5%, respectively) (Table 4).

**Table 4:** Withdrawals by reason, in the 12- and 24-month cohorts

Withdrawal Reason	Withdrawals in 12-month cohort n (%)	Withdrawals in 24-month cohort n (%)
Workload/work commitments	2438 (35.8)	879 (41.1)
Moving sites/leaving NHS	1303 (19.1)	491 (23)
Medical reasons	756 (11.1)	138 (6.5)
Logistical issues with attending appointments	622 (9.1)	203 (9.5)
Not stated	383 (5.6)	15 (0.7)
Other	316 (4.6)	51 (2.4)
Difficulties accessing testing	296 (4.3)	151 (7.1)
Personal reasons	189 (2.8)	80 (3.7)
Frequency of testing	153 (2.2)	37 (1.7)
Dislike testing methods	143 (2.1)	12 (0.6)
Ineligible	109 (1.6)	11 (0.5)
Management of results	47 (0.7)	9 (0.4)
Changes in attitudes towards the study over time	34 (0.5)	40 (1.9)
Implication of results	29 (0.4)	20 (0.9)
**Total**	**6,818 (100.0)**	**2,137 (100.0)**

### Participant engagement

In terms of participant engagement with the study, among those who completed 12-months of follow-up, 98.1% of participants completed at least one fortnightly follow-up survey in the 12-months (median number completed: 24; IQR: 16–26). Among those who completed 24-months of follow-up, 99.9% of participants completed at least one fortnightly follow-up survey in the 24-months (median number completed: 49; IQR: 38–52).

### Participant feedback survey

A total of 9,447 out of 32,845 participants (29%) completed the one-off feedback survey. Respondents were representative of the SIREN cohort, predominately female (*n* = 8,004, 85%), with the highest percentage of respondents in the 35–44 age group (*n* = 3,635, 38%), Nursing staff group (*n* = 3,203, 34%) and from South-West England (*n* = 1338, 14%).

The SIREN study scored highly across capability, opportunity and motivation categories and an overview of key survey results can be found in Table 5. In terms of participant capability (an individual’s psychological and physical capacity to engage in the activity), 90% of respondents found the study easy to participate in, and 90% understood how their data was being used and what it was contributing to. Responses to opportunity categories (external factors that make a behaviour possible) were lower, with 66% reporting they were kept up to date about the study by their organisation and 70% agreeing that taking part in SIREN made them more aware of research at their organisation. SIREN scored highly across motivation categories (conscious and unconscious cognitive processes that direct and inspire behaviour): 93% agreed that SIREN made them feel like they were making a valuable contribution to the pandemic response; 87% agreed that participating reassured them about their COVID-19 status and 86% felt like a valued member of the study.

**Table 5:** Results of the SIREN participant feedback survey

Feedback statement	Agree	Neutral	Disagree
	**n (%)**	**n (%)**	**n (%)**
**COM-B component: Capability**			
I know where to go to find information about the SIREN study	7801 (83.1)	1284 (13.7)	302 (3.2)
I know where to go to ask any questions I have about the SIREN study	7722 (82.5)	1293 (13.8)	345 (3.7)
I understand how my data is being used and what it is contributing to	8257 (90.0)	768 (8.4)	167 (1.8)
	**Easy**	**Neutral**	**Difficult**
	**n (%)**	**n (%)**	**n (%)**
I found the SIREN study…to participate in	8289 (90.2)	792 (8.6)	107 (1.1)
	**Agree**	**Neutral**	**Disagree**
	**n (%)**	**n (%)**	**n (%)**
**COM-B component: Opportunity**			
Being part of the SIREN study has made me more aware of research going on in my own organisation	6474 (70.3)	2245 (24.3)	482 (5.3)
I am kept up to date with information about the SIREN study by my organisation	6137 (65.6)	1862 (19.9)	1362 (14.5)
I am kept up to date with information about the SIREN study by the UKHSA SIREN study team	7866 (85.8)	1134 (12.4)	169 (1.8)
**COM-B component: Motivation**			
Participating in the SIREN study to date has made me more likely to participate in future research studies	7153 (77.7)	1965 (21.3)	86 (1.0)
Participating in the SIREN study makes me feel like I am making a valuable contribution to the COVID-19 pandemic response	8581 (93.4)	564 (6.1)	39 (0.5)
Being testing regularly as part of the SIREN study has made me feel more reassured about my COVID-19 status	8019 (87.2)	1028 (11.2)	151 (1.6)
I have felt like a valued member of the SIREN study	7896 (86.2)	1191 (13.0)	71 (0.8)

Analysis of open text responses to the statement *“I have felt like a valued member of the SIREN study”* highlighted positive and negative factors influencing motivation.

Three positive themes emerged which are described below.

A first theme was that participants appreciated friendly and accommodating research teams. Examples included site research teams helping participants to fit SIREN testing around their working schedules and shift patterns.

*“The team are welcoming and even accommodate my working pattern so I can do the tests.”*

*“A very friendly team that makes you feel valued.”*

A second theme highlighted was the positive role of communication from the SIREN team. Responses included reference to thank you messages, in addition to communications highlighting how participant samples contributed to research efforts.

*“Regular communication from SIREN which always included a thank you. Have also joined/watched all of the webinars which have always stressed the value of every single test sample submitted.”*

*“It helps the global community get the relevant information …and hopefully prepare communities on dealing with other viruses.”*

A final theme was that participants felt valued because they perceived taking part in SIREN was contributing to pandemic response efforts.

*“The information provided has hopefully made a difference to the approach to managing and minimising the effect of COVID.”*

*“Seeing Chris Whitty quote results has really made me feel like I am contributing to something which directly relates to decisions made about wider society.”*

Three negative themes emerged as described below.

Participants who did not feel valued like valued members of the SIREN study highlighted that inflexibility in local study arrangements made participation challenging.

*“So unfortunately, the research unit where I work have become very inflexible and will only do swabs and bloods on the two days a week when I don’t work.”*

*“Was given no more than 48 hours’ notice for appointments.”*

Participants who did not feel valued flagged the importance of communication, citing a lack of response to enquiries and inconsistent sharing of results.

*“I didn’t receive the text messages promised and when I asked about this had no response.”*

*“I do not feel the results are clearly disseminated – although I see sporadic emails announcing a Webinar regarding this study, I do not feel or understand the benefit of being part of this study.”*

The lack of individualised test results received by participants was highlighted as a third theme.

*“There has been very little feedback from any of my tests. Simply being told that if a PCR was positive I’d be told, is less reassuring that being actively told each result when it was known.”*

Feedback from participants who did not feel valued highlighted the importance of communicating at all stages of the study, including site close out.

*“I couldn’t continue after the study closed at my site. I would have liked the opportunity to continue.”*

*“The way it ended abruptly with no prior warning or explanation made me feel undervalued.”*

### Participant feedback at withdrawal

Analysis of open text responses captured via the withdrawal survey demonstrates many participants who withdrew from the study had a positive experience (49%, *n* = 797), with 38% (*n* = 617) reporting a neutral experience and 12% (*n* = 202) reporting a negative experience. Analysis of responses using the COM-B framework provides further detail on factors impacting participant capability, opportunity and motivation to be a part of the study, with many similarities in themes between feedback from participants active in the study (above) and those who withdrew. Themes relating to opportunity and motivation were cited more frequently than capability. In particular, participants who withdrew valued the opportunity to contribute to the pandemic response and acknowledged the scientific importance of SIREN research as motivating factors. Negative factors included finding the study burdensome and the feeling dissatisfied with the individualised test result reporting available at their site.

### Site research team feedback

Two focus groups with attendees from SIREN site research teams took place to explore factors associated with participant retention. Twelve research team members representing 9 SIREN sites attended a focus group. Most participants were female (92%, *n* = 11), white (83%, *n* = 10) and all aged 35 or above.

Respondents described considerable variation in how SIREN was delivered locally across sites and how participant retention was managed.

A key observation was that participant motivation for remaining in the study changed as the pandemic progressed, and local teams had to adapt their retention strategies in response. Initially, many SIREN participants reported that their primary motivation for taking part in SIREN was not the individual protection it provided, but the ability to contribute to the evidence base about COVID-19. As this was a novel disease, participants felt their contributions were particularly crucial to understand more about it and provide more certainty to wider society.

In addition, access to PCR and antibody testing was a motivator to join the study, as a means of helping keep families and patients safe (PCR), understanding their own immunity (antibody) and as an enabler for activities during a period of restrictions.

*“We had a lot of people that were quite interested at the beginning. Very keen, actually. We almost got overrun with it. And I think because there wasn’t any swabbing, there wasn’t lateral flows back then. The only thing you could do was have a PCR test, and they weren’t that easy to get, I seem to recall. So we had a lot of people that were very interested because simply the fact that they could assure the safety of their family every two weeks. That seems to be the big motivation. And I’m going to say, unashamedly, we played on that a little bit, and it worked.”*

*(SIREN site research team member, Focus Group 1)*

With the availability of Lateral Flow Devices (LFDs) to the general public, respondents observed that for some participants the value of PCR testing through SIREN was reduced. Changes in hospital staff testing policies and the introduction of policies for regular LFD testing also had consequences for SIREN participation.

*“The lateral flow that the trust mandated for all clinical staff took precedence, and people suddenly started to see the SIREN study as optional, which it is optional, of course. But they started to see it as, well I did my lateral flow yesterday so why do I need to bother doing a PCR today when my lateral flow was negative, so they stopped doing it that way.”*

*(SIREN site research team member, Focus Group 1)*

Factors perceived to impact participant motivation over time were primarily external to the study, such as reduced threat perception to SARS-CoV-2, the availability of SARS-CoV-2 testing outside the study, the introduction of COVID-19 vaccination, and changing pandemic control measures – with the removal of non-pharmaceutical interventions and increased social mixing over time. These external changes appeared to impact participant perceptions of the value of the study.

In addition to these external factors, respondents also identified challenges internal to the study, with participants dropping out of the study due to the physical discomfort of testing, the negative impact of having to take time-off work due to a positive test, and fatigue from attending so many SIREN appointments.

Focus group respondents described how they adapted the retention strategies over time in response to changing participant motivations. When asked for examples of what worked well, the following themes emerged.

#### Theme 1: share study findings with participants

Focus group respondents described how it was important to communicate study findings with participants. Examples were given where research teams had shared presentations of localised data with participants to demonstrate the wider value of their individual contribution. Research teams felt it was important to share this information as the initial reason for taking part was no longer fulfilled by SIREN alone and participants needed something more in return for their contribution to the study.

*“So then we changed our strategy a little bit, and we shared some of the information about the inputs of the study, some of the things that the preliminary stuff that started to come out, so the people could see that it was bigger than just them. And that actually it was really important that they kept contributing.”*

*(SIREN site research team member, Focus Group 1)*

#### Theme 2: convenience as a facilitator for retention

Focus group respondents considered that the consistency and physical location of SIREN activities had important implications for retention. Better engagement was reported when the local SIREN team (and clinic) was physically closer to the hospital. Reducing the amount of travel required and co-locating services was identified as helping with retention as it minimised the testing burden of time and distance travelled. Respondents described a range of retention strategies used across the different research sites to increase the convenience of study participation. This included flexible delivery models such as drop-in and fixed appointments to accommodate the needs of participant shift patterns and preferences, and timely communications with reminders and results as external prompts to remain engaged.

*“The pathology department is right by phlebotomy, so they could then go and get their blood test done. So it was actually only really one trip, and they could get everything done in that one area. And I think that helped with retention.”*

*(SIREN site research team member, Focus Group 2)*

#### Theme 3: incentives and tokens of appreciation as facilitators

Respondents discussed that incentives such as raffles and tokens of appreciation such as cupcakes, badges and certificates created opportunities for positive and engaging communications.

*“SIREN provided us with some certificates for all the participants as well as there were little badges that they could wear around... They were so inspired by it all, they were wearing it with pride and those who did not get one were complaining that they didn’t get. But it was very helpful to have that little bit of incentive.”*

*(SIREN site research team member, Focus Group 1)*

However, with the scale of SIREN and limited budget, opportunities for offering these were limited, and some respondents voiced frustration at the quality and quantity of tokens provided by the central team. While incentives were recognised as positive for motivation, research teams felt maintaining a relationship with participants was more important for retention and maximising participation.

## Discussion

SIREN was conducted under unique circumstances. Establishing SIREN as a large prospective cohort study, requiring sustained and frequent participant testing, in addition to fulfilling a role as an agile pandemic research response study requiring multiple adaptations over time, was ambitious. Evaluation of the cohort retention programme within this study has provided valuable insight into the acceptability of this study to participants, identifying factors that influenced retention, and has provided learning for future cohort studies, pandemic response studies, and studies involving the unique cohort that is HCWs.

In terms of methodological learning, retention in SIREN was very high, with similar proportions of participants completing the 12 month and 24-month cohorts. This finding supports the existing literature on cohort retention, which recommends a multifactorial approach [4] with strategies to reduce barriers to participation, build a study community and encourage follow-up [1], underpinned by key resources such as dedicated staff and data on retention collected over time [3–5].

In addition, using the COM-B model to analyse survey data has provided targeted next steps for the SIREN cohort retention programme and future iterations, demonstrating its usefulness and applicability to wider studies. For example, analysis of the participant feedback survey suggested factors influencing an individual’s opportunity to participate in the study required greater focus. This could include strengthening links between central and local study teams, to ensure local teams are equipped with the resources needed to keep participants up to date. This was supported by COM-B analysis of open text responses in the withdrawal survey, which suggested a higher frequency of responses relating to capability and motivation factors compared to opportunity.

Further lessons identified are highlighted in Table 6. Crucially, being able to understand and monitor participant motivation over time is key to cohort retention. Monitoring withdrawal data suggested the influence of external factors on retention, with peaks in withdrawals coinciding with high vaccine coverage in the cohort and with changes to testing policies (locally and nationally). However, identifying motivating factors at the point of withdrawal is too late, reinforcing the importance of gathering feedback from participants during follow-up, via feedback surveys, focus groups or the PIP. Informed by this, the SIREN study has since introduced more frequent participant feedback surveys.

**Table 6:** An overview of lessons identified to inform future pandemic response cohort studies

Theme	Learning
**1** Understand and monitor participant motivation	• Understanding participant motivation is crucial to maintaining retention • However, motivation changes over the course of the study and is influenced by internal and external factors. For SIREN, internal factors included study fatigue and external factors included the introduction of vaccines and rapid test results via LFDs • Retention programmes need to evolve to respond to changes in motivation
	**What works:** Obtaining regular feedback helps monitor changes in motivation to then adapt retention programmes, and this requires dedicated resource. For SIREN, the PIP have been crucial to understanding participant motivation by enabling a two-way conversation between researchers and participants. Other examples include introducing feedback surveys, providing a central mailbox for participants which is actively monitored, hosting regular meetings with site research teams who interact with participants more regularly.
**2** Provide inclusive and comprehensive communication	• Inclusive and comprehensive communications is a cornerstone of maintaining participant retention, but this can be overlooked in periods of high study activity • How much and what type of information to share, in addition to the method of communication, is cohort-specific and requires consideration
	**What works:** To allow for different communication preferences, SIREN has trialled a range of approaches including a newsletter, a dedicated webpage, blogs, videos, live webinars and email or SMS updates, seeking feedback from participants and the PIP along the way. Developing templates for communication can help reduce the time burden of communications. SIREN have developed digital newsletter and plain language summary templates which enable the quick delivery of study news.
**3** Acknowledge contributions	• Saying thank you throughout a participant’s study journey is essential to retention, as it can impact on motivation to continue and actively engage with the study • A pragmatic approach to acknowledging the contribution of participants can be included in pandemic response research
	**What works:** Due to resource constraints, SIREN has had to think creatively about how to thank participants over the course of the study. We have utilised newsletters, webinars and videos to circulate thank you messages. We have marked study anniversaries with certificates (postal or digital) and prize draws, and reached out to high profile individuals to feature in videos.
**4** Support collaboration between central and local teams	• Collaboration between central and local teams is essential to run a decentralised study because both are engaging with participants and have the potential to influence retention • SIREN was established at pace and scale, and this made it more challenging to provide sites with the guidance needed to ensure participants had a uniform study experience to encourage retention
	**What works:** SIREN introduced regular check-in meetings with site research teams to troubleshoot study or participant challenges. We have also used focus groups and interviews to obtain more detailed feedback from site teams. Setting out expectations and roles for each organisation as early as possible in the study is also helpful.
**5** Develop the skillset of research study team	• Participant retention requires dedicated resources and a specific skillset, and this can be overlooked in the design phase of research studies, particularly in a pandemic response
	**What works:** Designing and delivering study communications is a skill, and research teams need to consider whether to recruit for this specifically or develop workforce capability so that this responsibility is shared. The latter works well in studies where multiple research team members interact with participants. SIREN also invested in business support throughout the study, positioning them as essential team members. In particular this helped with monitoring the participant mailbox which takes up a considerable amount of time

Changing participant motivation can be responded to and addressed via study communications, which need to be inclusive and comprehensive. Keeping participants updated can be overlooked during periods of high study activity, which is why establishing regular and standardised forms of communication is key. For example, SIREN established regular branded newsletters, webinars and plain language summaries of key research papers. The acceptability of this is reflected in the participant feedback survey responses, and is a key theme highlighted in the focus groups with site research teams. In addition, acknowledging the contributions of participants was also highlighted by the focus groups, with incentives and tokens of appreciation identified as facilitators for retention.

In terms of pandemic learning, the results of this evaluation support undertaking research on HCWs during a pandemic response period. This is an important finding, as it is recognised that a focus on early warning and routine surveillance of specific high-risk population groups and settings is a key part of pandemic preparedness [16]. HCWs are the frontline of any outbreak or pandemic response, and research into this population group has the potential to offer early insight into: infection trends and transmissibility; vaccine effectiveness as a frequent priority group for vaccination; HCW illness or absence which can inform health service planning [17]; and the effectiveness of Infection Prevention Control (IPC) measures which aim to reduce the risk of transmission in hospital and social care settings [16, 18].

SIREN was conducted at scale to respond to an evolving pandemic, which necessitated numerous changes over time as the pandemic evolved. The study took a pragmatic approach to cohort retention and as such there are limitations to consider. The relatively low response rate to the participant feedback survey (29%, *n* = 9,447) and the small number of focus group participants (*n* = 12) are limitations of this study. A repeated feedback survey and focus groups would have been helpful to ensure the views collected are generalisable to the cohort overall. Questions in the participant feedback survey could have been expanded to capture views on individual cohort retention strategies. Further evaluation of individual SIREN site approaches to running the study and the impact this has on cohort retention could optimise understanding of factors enabling retention particularly given the frequency of references to local research teams in participant feedback responses.

## Conclusion

This robust mixed methods evaluation involving quantitative and qualitative analysis of cohort retention data has highlighted the challenges and learning from retaining the cohort of a large, multisite prospective cohort study established to contribute to a pandemic response. With a multi-method cohort retention programme, SIREN achieved high retention in the 12- and 24-month follow-up cohorts. We acknowledge some factors impacting retention were outside the control of the study, and the altruistic motivation of participants to contribute to the pandemic response cannot be underestimated. Participant feedback was positive overall, indicating that the study provided participants with the necessary capability, opportunity and motivation to remain in the study. Site feedback provided learning for future multicentre research studies, offering insights into key facilitators and common challenges faced. While there is no single agreed approach to cohort retention, the SIREN approach provides valuable learning for future cohort retention programmes, pandemic response studies and studies seeking to engage with healthcare worker cohorts.

## Supplementary Information

Supplementary Material 1.

## Data Availability

Anonymised data will be made available for secondary analysis to trusted researchers upon reasonable request.

## Abbreviations

COM-B: Capability, opportunity, motivation, behaviour
HCW: Healthcare workers
IPC: Infection, prevention, control
IQR: Interquartile range
LFD: Lateral flow device
NHS: National Health Service
NIHR: National Institute for Health and Care Research
PIP: Participant involvement panel
PCR test: Polymerase chain reaction test
SIREN: SARS-Cov2 Immunity & Reinfection Evaluation study
UKHSA: UK Health Security Agency
