# Supplementary material for: Cohort retention in a pandemic response study: lessons from the SARS-CoV2 Immunity & Reinfection Evaluation (SIREN) study
Source: BMC Med Res Methodol. 2025 Jan 30;25:27. doi: 10.1186/s12874-025-02469-6 (PMC11783804; doi:10.1186/s12874-025-02469-6)
Supplement: Supplementary file 1 — Supplementary Material 1. [file 12874_2025_2469_MOESM1_ESM.docx]

**Appendix 1.** Participant feedback survey questions

We are reflecting on the overall experience of SIREN participants to date. Please take a

moment to think back over your SIREN study journey so far – including how you first heard

about the study, the appointments you have attended to date and the communications you

have received from your site and UKHSA. We would be grateful if you could rate the

statements below.

1. Participating in the SIREN study to date has made me more likely to participate in future research studies.
2. Being part of the SIREN study has made me more aware of research going on in my own organisation.
3. Participating in the SIREN study makes me feel like I am making a valuable contribution to the COVID-19 pandemic response.
4. Being tested regularly as part of the SIREN study has made me feel more reassured about my COVID-19 status.
5. I have felt like a valued member of the SIREN study.
6. Please use the space below to describe why or why not.
7. I know where to go to find information about the SIREN study.
8. I know where to go to ask any questions I have about the SIREN study.
9. I am kept up to date with information about the SIREN study by my organisation.
10. I am kept up to date with information about the SIREN study by the UKHSA SIREN study team.
11. I found the SIREN study…to participate in.
12. I understand how my data is being used and what it is contributing to.

Thank you for completing this survey. Please ensure you click submit for this survey to be processed.
